# P2Y_2_ receptor knock-out mice display normal NaCl absorption in medullary thick ascending limb

**DOI:** 10.3389/fphys.2013.00280

**Published:** 2013-10-09

**Authors:** Rita D. Marques, Helle A. Praetorius, Jens Leipziger

**Affiliations:** Department of Biomedicine, Aarhus UniversityAarhus, Denmark

**Keywords:** purinergic, renal transport, loop of henle, P2Y_2_ receptors, NKCC2

## Abstract

Local purinergic signals modulate renal tubular transport. Acute activation of renal epithelial P2 receptors causes inhibition of epithelial transport and thus, should favor increased water and salt excretion by the kidney. So far only a few studies have addressed the effects of extracellular nucleotides on ion transport in the thick ascending limb (TAL). In the medullary thick ascending limb (mTAL), basolateral P2X receptors markedly (~25%) inhibit NaCl absorption. Although this segment does express both apical and basolateral P2Y_2_ receptors, acute activation of the basolateral P2Y_2_ receptors had no apparent effect on transepithelial ion transport. Here we studied, if the absence of the P2Y_2_ receptor causes chronic alterations in mTAL NaCl absorption by comparing basal and AVP-stimulated transepithelial transport rates. We used perfused mouse mTALs to electrically measure NaCl absorption in juvenile (<35 days) and adult (>35 days) male mice. Using microelectrodes, we determined the transepithelial voltage (V_te_) and the transepithelial resistance (R_te_) and thus, transepithelial NaCl absorption (equivalent short circuit current, I'_sc_). We find that mTALs from adult wild type (WT) mice have significantly lower NaCl absorption rates when compared to mTALs from juvenile WT mice. This could be attributed to significantly higher R_te_values in mTALs from adult WT mice. This pattern was not observed in mTALs from P2Y_2_ receptor knockout (KO) mice. In addition, adult P2Y_2_ receptor KO mTALs have significantly lower V_te_values compared to the juvenile. No difference in absolute I'_sc_ was observed when comparing mTALs from WT and KO mice. AVP stimulated the mTALs to similar increases of NaCl absorption irrespective of the absence of the P2Y_2_ receptor. No difference was observed in the medullary expression level of NKCC2 in between the genotypes. These data indicate that the lack of P2Y_2_ receptors does not cause substantial differences in resting and AVP-stimulated NaCl absorption in mouse mTAL.

## Introduction

The term purinergic signaling describes the cellular effects mediated by binding of extracellular nucleotides and nucleosides as local signaling molecules to adenosine or to P2 receptors. Nucleotides bind to P2 receptors, which are divided into G-protein coupled receptors (P2Y receptors) and ligand-gated ion channels (P2X receptors). Purinergic signaling and modulation of cell and organ function is ubiquitous involving most areas of physiology and pathophysiology (Burnstock, 2007). In renal epithelia, P2 receptors are expressed in all nephron segments and found both in the apical and basolateral membranes (for review see Rieg and Vallon, 2009; Praetorius and Leipziger, 2010). The renal epithelial cell has been described to release nucleotides under various conditions and is viewed as the primary source of extracellular ATP for paracrine signaling in the kidney (Odgaard et al., 2009; Praetorius and Leipziger, 2010). ATP is also found in trace amounts in the urine (Rieg et al., 2007; Contreras-Sanz et al., 2012). Functionally, extracellular nucleotides inhibit ion transport in the different renal tubular segments. Specifically, in the proximal tubule, ATP decreases of HCO^−^_3_ reabsorption (Bailey, 2004). In the collecting duct (CD), the epithelial sodium channel (ENaC)-dependent Na^+^ absorption (Lehrmann et al., 2002; Shirley et al., 2005; Pochynyuk et al., 2008) and ROMK channel-dependent K^+^ secretion is inhibited (Lu et al., 2000). Moreover, extracellular nucleotides reduce the H_2_O absorption via Aquaporin-2 (AQP2) in the inner medullary collecting duct (IMCD) (Kishore et al., 2009). In the thick ascending limb (TAL), extracellular ATP mediates an acute decrease in the transepithelial NaCl transport via basolateral P2X receptors (Silva and Garvin, 2009; Marques et al., 2012). Overall, purinergic signaling results in inhibition of water and/or salt transport in the kidney. It is currently viewed that intrarenal purinergic signals tonically dampen absorption and thus, act as an endogenous diuretic system (Praetorius and Leipziger, 2010).

The TAL is responsible both for the generation of the cortico-medullary osmotic gradient and the dilution of the urine. Absorption of Na^+^ and Cl^−^ in TAL cells requires normal function of various ion transporters and channels. Na^+^ and Cl^−^ are initially transported from the tubular lumen by the apical Na^+^, K^+^, 2Cl^−^ cotransporter (NKCC2). Na^+^ leaves the cytosol via the basolateral Na^+^, K^+^-ATPase, whereas Cl^−^ passes through the basolateral chloride channel Kb (ClC-Kb) into the interstitium following its electro-chemical gradient. The K^+^ taken up by NKCC2 is recycled through apical ROMK channels. This active transport process produces a lumen-positive transepithelial voltage, which drives the paracellular transport of Na^+^, Ca^2+^, and Mg^2+^. Measurements of the transepithelial electrical parameters permits quantification of transepithelial NaCl transport (Greger, 1985).

In addition to basolateral P2X receptors (Marques et al., 2012), the mouse TAL cells expresses P2Y_2_ receptors in both apical and basolateral membranes (Paulais et al., 1995; Jensen et al., 2007). However, there is currently no evidence for a direct P2Y_2_ receptor-mediated modulation of the transepithelial ion transport in this tubular segment. We recently described that acute stimulation of the basolateral P2Y_2_ receptor with UTP (100 μM) had no apparent effect on Na^+^ and Cl^−^ absorption in the isolated perfused mTAL (Marques et al., 2012). The present study was conducted to clarify a potential chronic effect of P2Y_2_ receptor deficiency on NaCl absorption, specifically in the mTAL. To evaluate any changes on NaCl transport, the technique of isolated perfused mTAL tubules was used to determine electrogenic Na^+^ reabsorption. The main finding of this study is that the resting or AVP-stimulated NaCl absorption is similar in P2Y_2_ WT and KO mice. Thus, the P2Y_2_ receptor appears to play no major role in the regulation of mTAL NaCl reabsorption.

## Materials and methods

### Animals

In this study, 3.5–8 weeks old male mice of mixed genetic background (B6D2/SV129) were used. The P2Y_2_ receptor knockout (KO) mice were bred from heterozygous families and genotyped as described previously (Matos et al., 2005). Animals had free access to standard rodent chow and tap water. The mice were bred in house and handled according to Danish animal welfare regulations.

### Tubule perfusion and measurement of ion transport in mTAL

**Figure 1:**
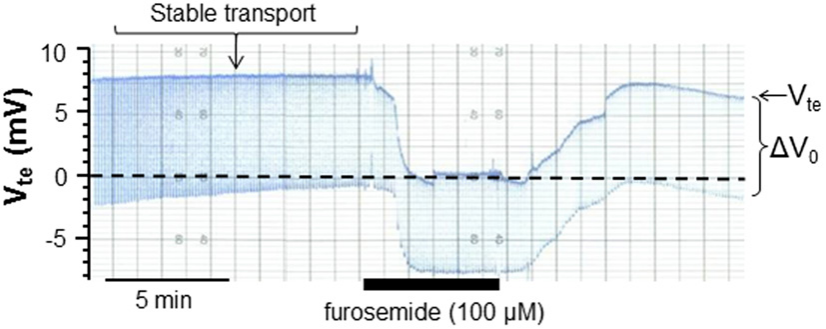
**Original recording of the lumen-positive transepithelial voltage (V_te_) and voltage deflections (Δ *V*_0_) in an isolated, perfused mouse medullary thick ascending limb (mTAL).** Steady state conditions were reached after about 15 min. The arrow indicates the time point used to quantify transport. In addition, the reversible blocking effect of luminal furosemide (100 μ M) is shown.

Mice were sacrificed by cervical dislocation. The kidneys were removed and placed in ice-cold control solution (see below) before slicing and dissected as described before (Wright et al., 1990). The dissection of mTAL tubules from the inner stripe of the outer medulla was performed in control solution (see below) placed in a chamber cooled to 4°C using fine watchmaker forceps. The isolated nephron segment was transferred to a perfusion chamber at 37°C on an inverted microscope and perfused by a system of concentric glass pipettes (Greger and Hampel, 1981) leaving the opposite end of the tubule open. Ion transport in isolated perfused tubules was measured as previously described using a double-barreled perfusion pipette (Lehrmann et al., 2002). In summary, the transepithelial voltage difference (V_te_) was measured via one barrel between the lumen and a reference electrode placed in the bath. Through the other barrel a small current pulse of 38.7 nA was injected into the tubular lumen and measured as a voltage deflection (Δ*V*_0_). The length and the diameter of the tubules were documented with transmitted light microscopy images to quantify the tubule's cable properties. The cable equation was then used to determine the resistance of the tissue (R_te_ in Ω · *cm*^2^). Applying Ohm's law, the equivalent short circuit current (I'_sc_ in μA/cm^2^) was then calculated as a measure of NaCl transport in the mTAL. In Figure 1 an original trace with the used protocol is shown. After mounting the mTAL a stable lumen-positive V_te_ was reached within some 10–15 min. Transport was quantified after stable transport conditions were reached for a period of 10 min. The arrow in Figure 1 shows the time point from which the V_te_ and R_te_ values were used. The figure also displays an original effect of luminal furosemide (100 μM) indicating complete and reversible inhibition of the transport voltage.

### Western blotting

Age matched (44–45 days) male WT and KO mice were anesthetized with isoflurane, surgically opened and the left ventricle perfused with 20 ml PBS to rinse all blood from the kidneys. The mice were than sacrificed and the kidneys removed. The inner stripe of the outer medullar was dissected from both kidneys and homogenised with a plastic pestle in ice cold 150 μl lysis buffer (200 mM mannitol, 80 mM HEPES, 41 mM KOH, pH 7.5) supplemented with protease and phosphatase inhibitor cocktails (cOmplete ULTRA Roche, Switzerland). Homogenates were then spun (4.000 rpm, 15 min, 4°C). Homogenisation and centrifugation was performed three times. Supernatants (150 μl) were subjected to ultracentrifugation (20.800 *g*, 1 h, 4°C). Pellets (membrane fraction) were dissolved in 50 μl lysis buffer. Protein concentration was determined with a Pierce® BCA Protein Assay Kit (Pierce Biotechnology, USA).

Proteins were separated by 7.5% SDS-PAGE before transferring them to PVDF-plus transfer membranes (MSI, USA). Membranes were blocked with non-fat dried milk (blocking grade blocker, Biorad) overnight at 4°C. Primary antibody incubation was done for 1 h 40 min at room temperature (1:4000 in 5% BSA PBS/0.5% Tween). The polyclonal NKCC2 antibody was a kind gift from J. Loffing, Institute of Anatomy, University of Zürich, Switzerland. Equal lane loading of the protein samples was documented by β -actin detection (Pan Actin AB #4968, Cell Signaling Technology, USA). Blots were incubated with horse-radish peroxidase-conjugated anti-rabbit secondary antibody for 1h at room temperature. Membranes were washed after both antibody incubations (3 × 10 min in PBS/0.5% Tween and once with PBS 5 min). Blots were analyzed with an enhanced chemiluminescence detection system (FPM-100A, GE Healthcare, USA). Densitometry was performed with the ImageJ (NIH, USA) software.

### Solutions and chemicals

Tubule perfusion experiments were performed at 37°C with the following control solution (in mM): 145 NaCl, 1 MgCl_2_, 1.3 Ca-gluconate, 5 D-glucose, 0.4 KH_2_PO_4_, 1.6 K_2_HPO_4_, 5 HEPES. Solutions were titrated with NaOH to pH 7.4. All chemicals were obtained from Sigma-Aldrich Denmark (Vallensbaek, Denmark) and Merck (Darmstadt, Germany).

### Statistics

The data is shown as mean and standard error of the mean (mean ± sem). For experimental series *n* reflects the number of tubules used. On average 2 tubules were used from each mouse. Comparison of linear regression slope differences was performed in GraphPad Prism (vers. 4.02). Normal distribution was confirmed by the Kolmogorov-Smirnov test. Student's *t*-test was used in normal distribution series while for non-normal distributed series the Mann-Whitney test was used to compare mean values. A *p*-value of <0.05 was accepted to indicate statistical significance.

## Results

### Baseline transport properties of mTAL from p2y_2_ receptor WT and KO mice

**Figure 2:**
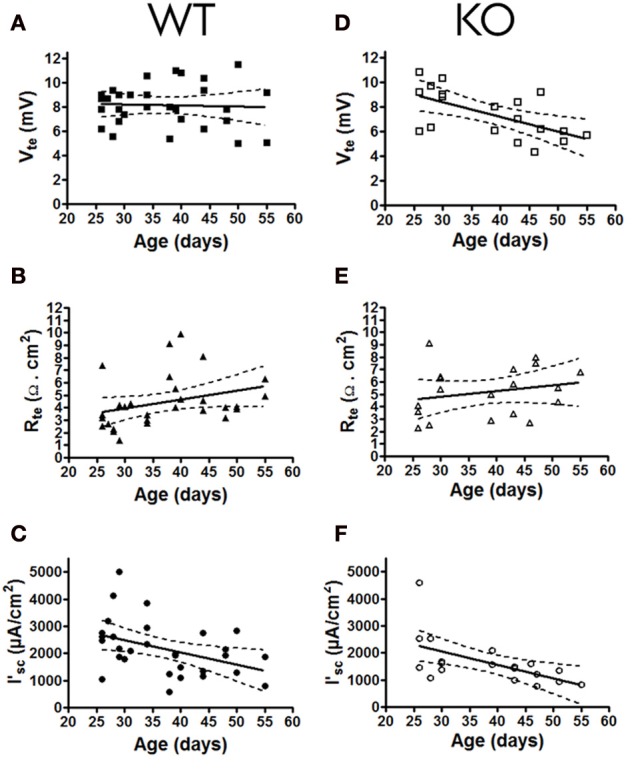
**Basal transport values (V_te_, R_te_, and I'_sc_) of mouse mTALs plotted as a function of age (in days) of P2Y_2_ receptor WT (A, B, C; *n* = 30) and KO (D, E, F; *n* = 19) mouse.** All 6 data panels include regression line fits where the stippled lines indicate the 95% confidence intervals.

**Figure 3:**
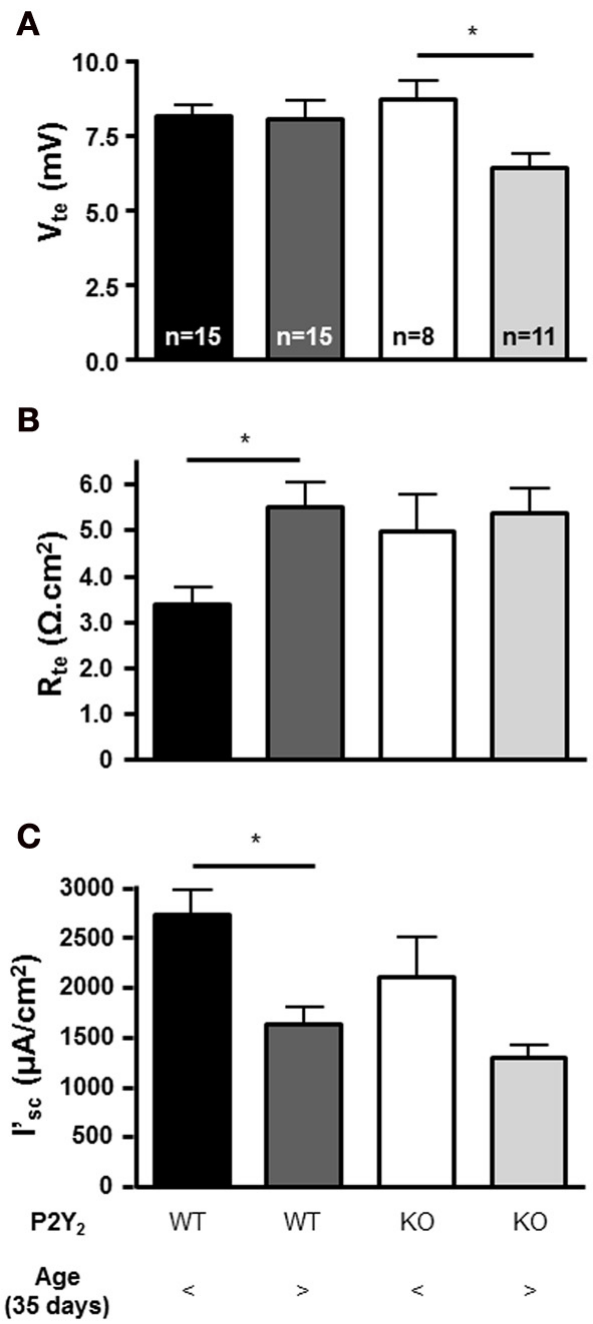
Ion transport parameters in mTALs (A: V_te_, B: R_te_, and C: I'_sc_) from juvenile (25–35 days) and adult (35–55 days) P2Y_2_ receptor WT and KO mice (^*^statistical significance *p* < 0.05).

To determine if the lack of the P2Y_2_ receptors influences the basal epithelial transport, we determined the steady state transepithelial transport parameters (V_te_, R_te_ and the derived I'_sc_) in isolated perfused mouse mTALs from WT and KO mice. All baseline transport values were plotted as a function of mouse age (Figure 2). This revealed a novel aspect of electrical transport data in mouse mTAL. In WT mice the V_te_ values appear stable, whereas the R_te_ values apparently increase with age. As a consequence of this, the calculated I'_sc_ values in older mice appear lower when compared to younger mice. Thus, this non-homogeneous data set precludes the direct comparison of the whole population from WT and KO mice. To test for possible differences between transport rates in mTAL from WT and KO mice, we divided the mice into two groups. Mice younger than 35 days are still considered juvenile and first viewed as adults after sexual maturation, which occurs around 35 days of age (Safranski et al., 1993). Figure 3 shows the grouped data, one with juvenile mice (25–35 days) and the other with adult mice (36–55 days). The adult WT mice show a significant increase in R_te_ as compared to juvenile mice (*p* = 0.0027). This results in a significantly lower Na^+^ and Cl^−^ absorption in the mTAL of adult WT mice (*p* = 0.0014). Interestingly, this ontogenetic effect on R_te_ is not seen in P2Y_2_ receptor KO mice.

Surprisingly, in mTALs isolated from KO mice the V_te_ values clearly decreased with age. In contrast, the V_te_ in WT mice did not alter as a function of age (Figures 2A,D, significantly different regression lines, *p* = 0.046). Thus, KO tubules of adult mice showed a significantly decreased V_te_ as compared with tubules from juvenile KO mice (Figure 3). Subsequently, we compared mTAL transepithelial transport data from juvenile WT with juvenile KO mice and adult WT with adult KO mice, which did not reveal any statistical significant differences (Figure 3).

In summary, these data report that (1) mTALs from adult WT mice have significantly lower NaCl absorption rates based on significantly higher R_te_values compared to tubules isolated from juvenile mice. This pattern was not observed in mTALs from KO mice. (2) mTALs from adult KO mice have significantly lower V_te_ values, which leads to a lower but non-significant (*p* = 0.08) reduction of NaCl absorption. (3) No difference was observed between tubules from WT and KO mice when comparing the absolute I'_sc_ data. Importantly, these data do not support higher NaCl absorption rates in the mTAL of P2Y_2_ receptor KO mice as suggested by elevated NKCC2 protein expression, which was previously documented in these mice (Rieg et al., 2007; Zhang et al., 2011).

### Expression of NKCC2 protein in mouse p2y_2_ WT and KO mTALs

**Figure 4:**
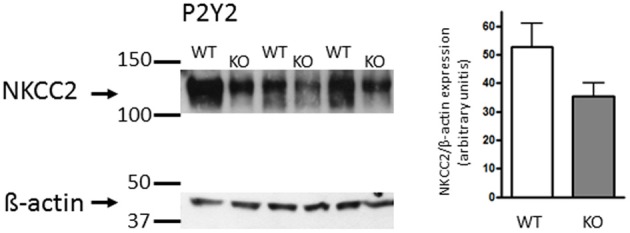
**Western blot revealing similar expression of NKCC2 in P2Y_2_ receptor WT and KO (*n* = 3 each) inner stripe of outer medulla kidney tissue.** Right side: The NKCC2 densitometric analysis is shown in reference to the equal dye loading control with β -actin.

Subsequently, we also performed the analysis of NKCC2 protein expression in membrane fractions from the inner stripe of the outer medullar (ISOM) in 3 P2Y_2_ WT and 3 P2Y_2_ KO mice. The data are depicted in Figure 4. To our surprise, we found no apparent up-regulation of NKCC2 protein in ISOM of P2Y_2_ KO mouse kidney. Thus, these data are in contrast to those reporting up-regulation of NKCC2 protein expression in P2Y_2_ KO mice (Rieg et al., 2007; Zhang et al., 2011).

### Avp-stimulated nacl transport in mtals frome p2y_2_ receptor WT and KO mice

**Figure 5:**
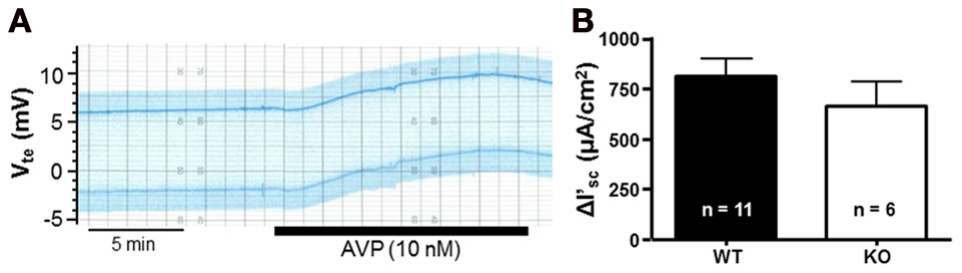
**AVP-stimulated NaCl transport increases in P2Y_2_ receptor WT and KO mTALs. (A)** An original recording of the AVP-induced (10 nM) NaCl transport stimulation, in a P2Y_2_ receptor WT mouse mTAL. **(B)** AVP-stimulated NaCl transport was studied in P2Y_2_ receptor WT (*n* = 11) and KO mice mTALs (*n* = 6). Summary of this results is presented as transport changes (± I'_sc_) in NaCl transport.

NaCl transport in the mTAL can be stimulated with hormones, such as AVP. This can be observed by a marked increase of V_te_ values after AVP addition in isolated perfused mTALs (Wittner et al., 1988). In this series of experiments, we studied the AVP-stimulated activation of NaCl transport in mTALs from P2Y_2_ receptor WT and KO mice to determine, if the lack of this purinergic receptor may have influenced the tissues functional responsiveness. An original experiment in a tubule from a WT mouse is shown in Figure 5A. In this original experiment, addition of basolateral AVP (10 nM) induced a slow (within minutes) increase of V_te_ from +6 mV to +9.5 mV reaching stable maximum values after about 10 min. The mean V_te_ increase in mTALs from WT mice was 2.5 ± 0.33 mV (*n* = 11), whereas the mean V_te_ increase in mTALs from KO mice was 2.2 ± 0.38 mV (*n* = 6). In Figure 5B, this transport activation is depicted for the entire series and shows an increase in I'_sc_ of 814 ± 86 μA/cm^2^ in mTALs WT mice (*n* = 11). In mTALs from KO mice AVP induced similar increases of transport that amounted to 667 ± 117 μA/cm^2^ (*n* = 6). These results indicate that the absence of the P2Y_2_ receptor has no apparent effect on AVP-induced transport activation in mouse mTAL.

## Discussion

The P2Y_2_ receptor is important in the regulation of transepithelial transport in the distal convoluted tubule (DCT) and CD. The acute stimulation of the receptor inhibits both Na^+^ and Ca^2+^ reabsorption in the DCT (Koster et al., 1996), ENaC-dependent Na^+^ reabsorption in the cortical CD (Lehrmann et al., 2002) and water reabsorption in the IMCD (Kishore et al., 2009). In the TAL, the P2Y_2_ receptor was found in the apical and basolateral membranes, and stimulation of these receptors on either side triggered an increase of [Ca^2+^]_i_ (Paulais et al., 1995; Jensen et al., 2007). The flow-induced increase of [Ca^2+^]_i_ in mouse mTAL was found to be mediated by the activation of these apical and basolateral P2Y_2_ receptors (Jensen et al., 2007). However, it is currently not understood, which biological role this receptor may serve in this nephron segment. Insulin is shown to enhance transepithelial transport in this segment in a Ca^2+^-dependent fashion (Ito et al., 1995). Since the P2Y_2_ receptor stimulation causes significant increments in [Ca^2+^]_i_, a straightforward hypothesis would be that the P2Y_2_ receptor would stimulate transport. However, in a previous study, we did not find any effect of acute addition of basolateral UTP on the transepithelial electrical parameters in mouse mTAL (Marques et al., 2012). This finding is substantiated by another study, where the oxygen consumption was used as a measure of TAL transport activity, which also failed to show major effects of P2Y_2_ receptor activation on the transport (Silva and Garvin, 2009). Interestingly, two independent studies have shown a substantial increase in medullary but not cortical NKCC2 protein expression in the TAL of P2Y_2_ receptor KO mice compared to WT (Rieg et al., 2007; Zhang et al., 2011, 2013). It was therefore likely to assume that NaCl absorption could be increased in mTAL of P2Y_2_ receptor KO mice. This notion was corroborated by data showing an increased furosemide-induced Na^+^ excretion in the P2Y_2_ receptor KO mouse (Rieg et al., 2007; Zhang et al., 2013). In this study we directly addressed whether the NaCl transport rates are actually increased in the KO animals as compared to controls. Surprisingly, we found that the resting I'_sc_ that quantitatively reflects the rate of NaCl absorption was not different in WT and KO mTALs. We therefore repeated the original protein expression experiments using the same NKCC2 antibody as used in the Rieg et al. paper (Rieg et al., 2007). Importantly, we cannot reproduce the results of an increased expression of NKCC2 protein in mTAL (Rieg et al., 2007; Zhang et al., 2011). Our experiments show no apparent difference in NKCC2 protein expression between P2Y_2_ WT and KO mouse ISOM. A likely explanation for this discrepancy may be found in the different genetic backgrounds of the P2Y_2_ knock-out mice used in the Rieg et al. and Zhang et al. paper as compared to our study. Whereas the former studies were conducted in mice on a C57BL/J6 and a B6D2 background our results were generated in mice bred on a mixed genetic (B6D2/SV129) background. In our study the unaltered NaCl transport rates therefore correlated with an apparently unaltered level of NKCC2 expression. The conclusion in our study that NKCC2 expression is not different has to be taken with some caution based on the limited number of 3 WT and 3 KO mice, respectively. When inspecting Figure 4, one could speculate that NKCC2 expression may be lower in our P2Y_2_ KO mixed genetic background mouse strain. Seen in conjunction with the transport data, the I'sc was tentatively but not significantly smaller in adult KO mouse mTAL as compared to adult WT mTAL (Figure 3C). Eventually, it should be interesting but also a tour de force to revisit this question by also using the P2Y_2_ receptor knock-out mouse back-crossed into the C57BL/J6 genetic background.

We extended this study to include measurement of transport after stimulation with AVP in WT vs. KO tubules. We found that AVP (10 nM) robustly stimulated NaCl absorption in all studied tubules independent of the presence of the P2Y_2_ receptor. In summary, we conclude that mTAL tubules from P2Y_2_ receptor deficient mice show intact resting and AVP-stimulated NaCl absorption, which is not different from WT controls.

Strictly speaking is it not correct to conclude that the transport properties of mTALs were similar in WT and KO mice. The transport of ions in the TAL also involves the paracellular route, which in this tubular segment is cation selective permitting transport of Na^+^, Ca^2+^, and Mg^2+^. The present study indisputably shows that the driving force for the paracellular transport, i.e., the V_te_ values, is similar in WT and KO mice. The permeability and the tightness of the paracellular shunt are critically dependent of the set of different claudins that are expressed in the epithelial barrier (Gunzel and Yu, 2013). In native respiratory epithelium, P2Y receptors have indeed been reported to acutely regulate of the paracellular shunt permeability (Poulsen et al., 2006). In this study, we were able to detect subtle ontogenetic differences in the R_te_ values in between the P2Y_2_ WT and KO mice, which may hint to a role of purinergic signaling in the regulation of the paracellular shunt. Moreover, did we detect lower V_te_ values in adult KO mice as compared to juvenile, a finding, which was not seen in the wild type (WT). It is thus fair to speculate that subtle transport differences may indeed exist in the P2Y_2_ receptor deficient mice.

In conclusion, the mTAL of P2Y_2_ receptor KO mouse shows an essentially normal transport function in terms of electrogenic transepithelial NaCl absorption during resting or AVP-stimulation. The normal mTAL function in P2Y_2_ KO mice of mixed genetic background correlated with an unaltered expression level of NKCC2.

### Conflict of interest statement

The authors declare that the research was conducted in the absence of any commercial or financial relationships that could be construed as a potential conflict of interest.
